# How to Live on One Dollar a Week

**Published:** 1902-02

**Authors:** 


### How to Live on One Dollar a Week.

The *Chicago Clinic,* in commenting upon an article written by Eustace A. Miles, entitled "How to Live Well and Keep in Training on One Dollar a Week," says: "We have no doubt that this would be valuable information for young physicians and an appended paragraph on 'How to Get the Dollar a Week' would add to the general value. From what we know of the cost of living, Mr. Miles' article would indicate that it should be in Mr. Bok's estimable journal of feminine vagaries rather than in a serious publication." R.

